# Socioeconomic variation in injury hospitalisations in Australian children ≤ 16 years: a 10-year population-based cohort study

**DOI:** 10.1186/s12889-018-6242-7

**Published:** 2018-12-04

**Authors:** Rebecca Seah, Reidar P. Lystad, Kate Curtis, Rebecca Mitchell

**Affiliations:** 1 Australian Institute of Health Innovation, Macquarie University, Sydney, NSW 2109 Australia; 2 Sydney Medical School, University of Sydney, Sydney, Australia

**Keywords:** Socioeconomic disadvantage, Childhood injury, Hospitalisation

## Abstract

**Background:**

Childhood injury remains a significant public health problem responsible for significant morbidity and mortality. However, injury has been found to increase with socioeconomic disadvantage for some injuries. The current study examines the 10-year epidemiological profile of injury hospitalisations of children ≤16 years by socioeconomic status for different age group and select types of injury.

**Method:**

A retrospective analysis of injury hospitalisations of children aged ≤16 years using linked hospitalisation and mortality records during 1 July 2002 to 30 June 2012 was conducted. Negative binomial regression was used to calculate incidence rate ratios (IRRs) for injury hospitalisation rates by socioeconomic disadvantage quintile.

**Results:**

There were 679,171 injury hospitalisations for children aged 0–16 years in Australia. Children in more disadvantaged socioeconomic quintiles were more likely to be hospitalised for an injury sustained by: assault (IRR range 1.40 to 3.64), poisoning (IRR range 1.29 to 1.36), heat and hot substances (IRR range 1.07 to 1.34), and pedestrian collisions (IRR range 1.06 to 1.54) than children in advantaged socioeconomic quintiles.

**Conclusions:**

Findings support the notion that the risk of injury hospitalisation among children differs according to socioeconomic gradient and has implications for childhood injury prevention. Policy makers should consider socioeconomic differences in the design of injury prevention measures, particularly measures directed at modifying the built environment and home-based interventions.

## Background

Globally, childhood injury is considered a substantial public health burden responsible for significant morbidity and mortality [1]. However, the burden of injury is not shared equally among all sub-populations, and generally it disproportionately affects those with a lower socioeconomic status (SES) [2]. Across several international studies, rates of injury mortality have been found to increase with socioeconomic disadvantage [3–5]. In comparison, studies examining the relationship between injury morbidity and SES are less consistent, with some reporting an inverse [4, 6], positive [7] or no relationship between SES and injury [8].

One explanation for this discrepancy may be that SES is differentially related to specific injury mechanisms [5]. The influence of socioeconomic disadvantage is not uniform across all injury types, and aggregating all injury may mask important differences [2]. For example, backyard swimming pool ownership and access to different types of recreational activities increases with more advantaged SES. Thus, the likelihood of sustaining an injury from a pool drowning and/or recreational activity would increase with individuals of a higher SES [9]. Conversely, pedestrian and/or motor vehicle collisions tend to be higher among those residing in lower SES communities, where the population density is higher and public safety measures such as safer road infrastructures may be lacking [10, 11].

The risk of unintentional and intentional injury also varies with age. For intentional injury, self-harm-related behaviours are noticeably higher in older children aged 12 to 16, [12, 13]. Conversely, hospitalisations due to burns, unintentional poisoning and drowning in the home occur more frequently in younger children under the age of seven [14]. To date, population-based studies focusing on the role of SES and risk of injury hospitalisations of children in Australia have been scarce. Given that socioeconomic disadvantage is a fundamental determinant of ill-health, an examination of its relationship with injury would inform prevention interventions based on socioeconomic gradient and injury type. The current study aims to examine the 10-year epidemiological profile of injury hospitalisations of children ≤16 years by SES for different age groups and select types of injury.

## Method

A retrospective analysis of injury hospitalisations for children aged ≤16 years using linked hospitalisation and mortality records during 1 July 2002 to 30 June 2012 was conducted. The method to obtain the hospitalisation and mortality data and to identify the injury hospitalisations has been described elsewhere [15] and is summarised here.

### Hospitalisation and mortality data

Hospitalisation information was obtained from the National Hospital Morbidity database and jurisdiction-based hospitalisation data collections in Australia. Data was available from all Australian states and territories, however in the Australian Capital Territory data were only available from 1 July 2004. Hospitalisation data includes information on patient demographics, source of referral, diagnoses, external cause, type of hospital separation (e.g. discharged, death), and place of occurrence. Diagnoses and external cause codes were classified using the International Classification of Diseases, 10th Revision, Australian Modification (ICD-10-AM) [16]. Injury admissions were identified using a principal diagnosis classification of injury (ICD-10-AM: S00-T78).

Hospitalisation and mortality records were probabilistically linked by the Australian Institute of Health and Welfare to enable identification of deaths after hospital discharge. Hospitalisation and mortality records for Western Australia were linked by the Western Australian Data Linkage Branch. The Tasmanian hospitalisation records were linked using a unique patient identifier, with mortality post-admissions recorded within Tasmanian hospitalisation data. In Victoria, 20.4% (*n* = 41,482) of the child injury hospital records were not able to be linked and included in the study due to incompleteness in the linkage variables, such as name and residential address.

### Identification of health conditions and injury severity

Chronic health conditions which are common among children (e.g., diabetes, asthma, cancer, obesity, cystic fibrosis) were identified within the hospitalisation data [17, 18]. A chronic health condition was defined as lasting up to ≥12 months, and placing a limitation on an individual’s ability for self-care, independent living, social interactions, and/or resulted in the need for ongoing healthcare service use [19]. Chronic health conditions were categorised as none, one, or two or more health conditions.

Injury severity was estimated using the International Classification of Disease Injury Severity Score (ICISS) by applying existing survival risk ratios (SRR) to injury diagnoses classifications in the hospitalisation data [20]. The ICISS was derived for each individual by multiplying the probability of survival for each injury diagnosis using SRRs derived for each injury diagnosis [20]. Three severity levels were used to define minor (≥0.99), such as concussive injury or fracture of the lower end of the humerus, moderate (> 0.941- < 0.99), such as fracture of the shoulder/upper arm or contusion of the abdominal wall, and serious (≤0.941), such as traumatic brain injury or fracture of the cervical vertebra [21].

### Identification of socioeconomic disadvantage

A measure of socioeconomic disadvantage for each hospitalisation was assigned to the child’s postcode of usual residence using the Index of Relative Socioeconomic Disadvantage (IRSD) [22]. The IRSD is an index of socioeconomic disadvantage, where lower scores indicate more disadvantaged areas. The IRSD’s quintiles are derived every five years from Australia’s population census using characteristics such as income, education, employment, occupation and other measures that indicate socioeconomic advantage (e.g., high income, tertiary education) and disadvantage (e.g., unemployment, low number of bedrooms in home). There were 7238 (1.1%) child injury hospitalisations where the IRSD was not available that were excluded from analyses.

### Data management and analyses

All statistical analyses were performed using SAS version 9.4 [23]. All hospital episodes of care related to the one injury event were linked to form a period of care. Denominator data for the number of children aged ≤16 years were obtained from the Australian Bureau of Statistics population estimates for each jurisdiction by IRSD [24]. Direct age-standardised incidence rates were calculated using the recommended Australian residential population at 30 June 2001 as the standard population [25, 26]. Due to changes in statistical area partitioning for IRSD quintiles in 2009, temporal trends for age-standardised hospitalisation rates were not statistically examined [27]. Thirty-day mortality was calculated from the date of admission of the index injury hospitalisation. Negative binomial regression analyses were used to calculate incidence rate ratios (IRRs) for injury hospitalisation rates by socioeconomic disadvantage quintile. The main explanatory variable in the models was the socioeconomic disadvantage quintiles, with age and sex entered as covariates and the log of the population as an offset.

## Results

Over the ten-year study period, there were 679,171 injury hospitalisations for children aged ≤16 years in Australia.

### Injury hospitalisation rates

**Fig. 1:**
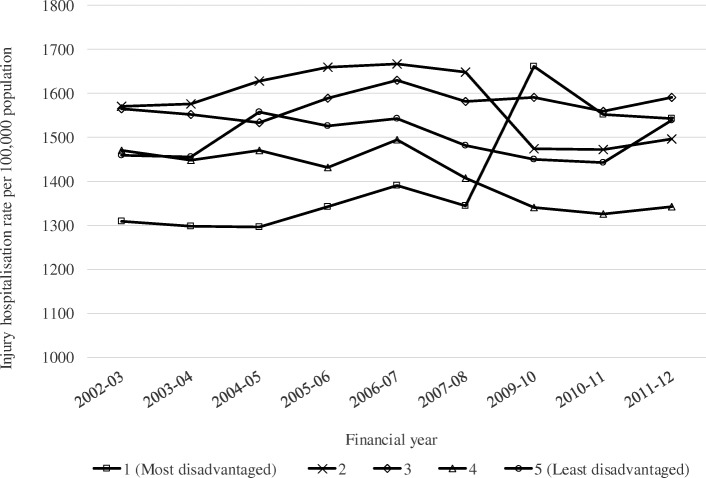
Incidence rates for hospitalisations in children ≤16 years by socioeconomic disadvantage quintile by financial year, linked hospitalisation and mortality records 1 July 2002 to 30 June 2012^1. 1^Hospitalisation rates exclude the Australian Capital Territory

The rates of injury hospitalisations remained relatively stable across each quintile between 2002 to 2008. Between 2008 and 2010, there was an increase in rates of injury hospitalisations for the most socioeconomically disadvantaged quintile, before declining between 2009 to 2011 (Fig. 1).

### Demographic and injury characteristics

Overall, males accounted for around two-thirds of injury hospitalisations. Children aged 11–16 years accounted for one in five hospitalised injuries across all SES disadvantage quintiles, while children living in the most disadvantaged SES quintile had the highest proportion of injury hospitalisations for serious injuries (8.2%). Thirty-day mortality was relatively similar for children living in each SES quintile (Table 1).

**Table 1:** Demographic characteristics injury-related hospitalisations by socioeconomic disadvantage quintile, linked Australian hospitalisation and mortality records, 1 July 2002 to 30 June 2012

	Most disadvantaged (n = 133,397)		2 (n = 141,812)		3 (n = 141,876)		4 (n = 126,287)		Least Disadvantaged (n = 135,799)	
	n	%	n	%	n	%	n	%	n	%
Gender^a^										
Male	84,434	63.3	90,659	63.9	90,921	64.1	80,132	63.5	86,239	63.5
Female	48,960	36.7	51,151	36.1	50,954	35.9	46,154	36.5	49,559	36.5
Age groups^b^										
0–5	47,550	35.6	46,438	32.7	47,480	33.4	44,589	35.3	46,928	34.6
6–10	32,312	24.2	34,770	24.5	34,770	24.5	31,252	24.7	34,662	25.5
11–16	53,535	40.1	60,379	42.6	59,626	42.0	50,446	39.9	54,209	39.9
Health conditions										
None	126,426	94.8	134,000	94.5	134,306	94.7	119,913	95.0	128,628	94.7
One	5924	4.4	6554	4.6	6413	4.5	5470	4.3	6235	4.6
Two or more	1047	0.8	1258	0.9	1157	0.8	904	0.7	936	0.7
Injury severity										
Minor	77,636	58.2	85,549	60.3	87,132	61.4	78,630	62.3	87,158	64.2
Moderate	44,766	33.6	45,974	32.4	45,078	31.8	39,361	31.2	40,935	30.1
Serious	10,995	8.2	10,289	7.3	9666	6.8	8296	6.6	7706	5.7
30-day mortality	250	0.2	228	0.2	189	0.1	169	0.1	127	0.1

^a^Information on gender was not known for 8 hospitalisations. ^b^Information on age for one hospitalisation was missing

### Injury mechanism

Fall-related injuries, and injury due to inanimate mechanical forces (such as being struck by an object) accounted for the highest proportion of child hospitalisations across each SES quintile. Child hospitalisations due to assault and poisoning injuries were highest for children living in the most disadvantaged SES quintile (Table 2).

**Table 2:** Injury mechanism for injury-related hospitalisations by socioeconomic disadvantage quintile, linked Australian hospitalisation and mortality records, 1 July 2002 to 30 June 2012

	Most disadvantaged (n = 133,397)		2 (n = 141,812)		3 (n = 141,876)		4 (n = 126,287)		Least Disadvantaged (n = 135,799)	
	n	%	n	%	n	%	n	%	n	%
Road transport	19,309	14.3	21,971	15.0	20,705	14.3	16,527	13.1	14,188	10.4
Pedestrian	1919	1.4	1626	1.1	1559	1.1	1284	1.0	1263	0.9
Pedal cyclist	6706	5.0	8160	5.6	8142	5.6	6883	5.5	7236	5.3
Motorcyclist	4459	3.3	5693	3.9	5268	3.6	3814	3.0	2276	1.7
Motor vehicle occupant^a^	3763	2.8	3382	2.3	3030	2.1	2441	1.9	1870	1.4
Other land transport	2462	1.8	3110	2.1	2706	1.9	2105	1.7	1543	1.1
Water, air and other and unspecified transport	412	0.3	510	0.4	500	0.3	500	0.4	580	0.4
Falls	46,662	34.9	51,905	36.6	54,332	38.3	50,711	40.2	57,263	42.2
Inanimate mechanical forces	24,392	18.3	24,682	17.0	25,344	17.5	22,125	17.5	23,144	17.0
Animate mechanical forces	7637	5.7	7637	5.3	8239	5.7	7213	5.71	7293	5.4
Drowning and submersion	583	0.4	583	0.4	532	0.4	534	0.4	512	0.4
Other threats to breathing	529	0.4	519	0.4	517	0.4	506	0.4	463	0.3
Electric current, radiation, extreme ambient air temperature and pressure	160	0.2	178	0.1	168	0.1	110	0.1	104	0.1
Smoke, fire and flames	1393	1.0	1026	0.7	867	0.6	674	0.5	480	0.4
Heat and hot substances	4225	3.2	3713	2.6	3083	2.1	3095	2.5	2905	2.1
Venomous animals and plants	1634	1.2	1727	1.2	1341	0.9	963	0.8	1029	0.8
Poisoning	5869	4.4	5470	3.8	5292	3.7	4485	3.6	4101	3.0
Intentional self-harm	3701	2.8	4067	2.8	3731	2.6	3174	2.5	3378	2.5
Assault	4399	3.3	3007	2.1	2643	1.8	1936	1.5	1535	1.1
Other and unspecified injury mechanism	12,492	9.4	14,264	9.9	14,582	10.1	13,734	10.9	18,824	13.9

^a^Includes heavy vehicle and bus occupants

### Adjusted incidence rate ratios

Children living in a more disadvantaged SES quintile were more likely to be hospitalised for an injury sustained by assault (IRR range 1.40 to 3.64), poisoning (IRR range 1.29 to 1.36), heat and hot substances (IRR range 1.07 to 1.34), and pedestrian collisions (IRR range 1.06 to 1.54) compared to children living in the least disadvantaged quintile (Table 3). The remaining injury mechanisms (i.e. pedal cyclists, falls, self-harm for 11–16 years), along with injuries that occurred in the home and farm, and injuries due to sports and leisure activities showed inconsistent relationships between SES disadvantage and injury. Overall, children in more disadvantaged SES quintiles were more likely to be hospitalised for a moderate or serious injury compared to the least disadvantaged quintile (Table 4).

**Table 3:** Adjusted incidence rate ratios for injury-related hospitalisations by quintile of socioeconomic disadvantage for select injury mechanisms, linked Australian hospitalisation and mortality records, 1 July 2002 to 30 June 2012^a^

Select injury mechanisms	IRR^b^ (95% CI)
Pedestrian	
Most disadvantaged quintile	1.54 (1.40 to 1.69)***
2nd	1.37 (1.24 to 1.50)***
3rd	1.30 (1.18 to 1.43)***
4th	1.06 (0.97 to 1.18)***
Least disadvantaged quintile	1.00
Pedal cycle	
Most disadvantaged quintile	0.91 (0.80 to 1.04)
2nd	1.13 (1.0 to 1.28)
3rd	1.14 (1.00 to 1.30)*
4th	0.96 (0.84 to 1.09)
Least disadvantaged quintile	1.00
Falls	
Most disadvantaged quintile	0.81 (0.75 to 0.88)***
2nd	0.96 (0.88 to 1.04)
3rd	0.99 (0.92 to 1.07)
4th	0.92 (0.85 to 1.00)*
Least disadvantaged quintile	1.00
Drowning – all ages	
Most disadvantaged quintile	1.10 (0.96 to 1.25)
2nd	1.22 (1.07 to 1.39)*
3rd	1.07 (0.94 to 1.22)
4th	1.06 (0.93 to 1.22)
Least disadvantaged quintile	1.00
Drowning (0–5 years)	
Most disadvantaged quintile	1.08 (0.75 to 1.55)
2nd	1.24 (0.86 to 1.78)
3rd	1.13 (0.78 to 1.63)
4th	1.13 (0.78 to 1.63)
Least disadvantaged quintile	1.00
Heat and other hot substances – all ages	
Most disadvantaged quintile	1.34 (1.23 to 1.46)***
2nd	1.33 (1.22 to 1.45)***
3rd	1.07 (0.98 to 1.17)
4th	1.11 (1.01 to 1.21)*
Least disadvantaged quintile	1.00
Heat and other hot substances (0–5 years)	
Most disadvantaged quintile	1.38 (0.99 to 1.92)
2nd	1.30 (0.94 to 1.81)
3rd	1.07 (0.77 to 1.50)
4th	1.09 (0.78 to 1.51)
Least disadvantaged quintile	1.00
Poisoning – all ages	
Most disadvantaged quintile	1.41 (1.25 to 1.59)***
2nd	1.33 (1.18 to 1.50)***
3rd	1.29 (1.14 to 1.45)***
4th	1.11 (0.98 to 1.25)
Least disadvantaged quintile	1.00
Poison (0–5 years)	
Most disadvantaged quintile	1.36 (0.95 to 1.95)
2nd	1.33 (1.18 to 1.50)***
3rd	1.29 (1.14 to 1.45)***
4th	1.11 (0.98 to 1.25)
Least disadvantaged quintile	1.00
Self-harm (11–16 years)	
Most disadvantaged quintile	0.92 (0.59 to 1.42)
2nd	1.16 (0.73 to 1.82)
3rd	1.19 (0.75 to 1.90)
4th	1.05 (0.66 to 1.68)
Least disadvantaged quintile	1.00
Assault	
Most disadvantaged quintile	3.64 (3.16 to 4.19)***
2nd	2.46 (2.13 to 2.84)***
3rd	2.06 (1.79 to 2.38)***
4th	1.40 (1.20 to 1.62)***
Least disadvantaged quintile	1.00

^a^Adjusted for age group, sex and socioeconomic status. ^b^The least disadvantaged quintile was used as the reference group for all variables. *p < 0.5, **p < 0.001, ***p < 0.0001

**Table 4:** Adjusted incidence rate ratios for injury-related hospitalisations by quintile of socioeconomic disadvantage for select place of occurrence, activity at time of injury and injury severity, linked Australian hospitalisation and mortality records, 1 July 2002 to 30 June 2012^a^

Select activity at time of injury, place of occurrence, and injury severity	IRR^b^ (95% CI)
Activity at time of injury	
Sport and leisure	
Most disadvantaged quintile	0.80 (0.66 to 0.96)*
2nd	0.92 (0.78 to 1.12)
3rd	0.99 (0.82 to 1.20)
4th	0.98 (0.81 to 1.18)
Least disadvantaged quintile	1.00
Place of occurrence	
Home	
Most disadvantaged quintile	1.16 (1.11 to 1.22)***
2nd	1.28 (1.22 to 1.34)***
3rd	1.18 (1.12 to 1.23)***
4th	1.06 (1.01 to 1.11)*
Least disadvantaged quintile	1.00
Farm	
Most disadvantaged quintile	3.64 (3.18 to 4.18)***
2nd	4.76 (4.17 to 5.44)***
3rd	3.69 (3.23 to 4.23)***
4th	1.92 (1.66 to 2.22)***
Least disadvantaged quintile	1.00
Injury Severity	
Minor (ICISS ≥0.99)	
Most disadvantaged quintile	0.89 (0.84 to 0.94)***
2nd	1.03 (0.97 to 1.08)
3rd	1.04 (0.99 to 1.09)
4th	0.94 (0.89 to 0.99)*
Least disadvantaged quintile	1.00
Moderate (ICISS > 0.941 – < 0.99)	
Most disadvantaged quintile	1.09 (1.03 to 1.15)*
2nd	1.17 (1.11 to 1.23)***
3rd	1.14 (1.08 to 1.20)***
4th	0.99 (0.94 to 1.05)
Least disadvantaged quintile	1.00
Serious (ICISS ≤0.941)	
Most disadvantaged quintile	1.43 (1.33 to 1.54)***
2nd	1.40 (1.31 to 1.51)***
3rd	1.31 (1.22 to 1.41)***
4th	1.13 (1.05 to 1.21)*
Least disadvantaged quintile	

^a^Adjusted for age group, sex and socioeconomic status. ^2^The least disadvantaged quintile was used as the reference group for all variables. *p < 0.5, **p < 0.001, ***p < 0.0001

## Discussion

This study identified that the incidence of injury hospitalisations due to pedestrian collisions, assault, poisoning, and heat and hot substances increased with SES disadvantage. These findings are consistent with the broader context of SES disadvantage as a primary risk factor for paediatric injuries [28, 29]. For example, children in the United Kingdom who resided in more deprived areas were up to four times more likely to be injured from a pedestrian incident than children in lesser deprived areas, with similar findings reflected in the United States, Canada and Sweden [28].

Relative SES disadvantage had the strongest relationship with assault-related injuries in the current study. Assault-related injury in all ages has been consistently found to be positively related to deprived populations, where poverty and income inequality tends to be higher [30]. However, for other intentional injury hospitalisations such as self-harm, there was an inconsistent relationship with SES disadvantage among 11–16 year olds, which directly contrasts with previous research where the role of SES deprivation using both individual (e.g., low parental education and household income) and area-based indicators were positively associated with risk of self-harm in adolescents [31, 32].

Children in more disadvantaged SES quintiles were found to be at a higher risk of poisoning, particularly those aged 0–5 years. Similarly, a Canadian study identified that children living in the most socioeconomically disadvantaged area in Quebec had a 68% increased risk of poisoning compared to children living in the least socioeconomically disadvantaged area [33]. Likewise, an increased risk of poisoning among 0–4 year olds was found with increasing social deprivation in England [34].

Despite accounting for the highest proportion of injury hospitalisations, fall injury hospitalisations did not show a consistent relationship with SES disadvantage. This study supports findings from a Swedish study which found no association between material deprivation, SES and falls [35]. Other area-based studies examining the role of SES and childhood falls have also showed mixed findings, with rates of hospitalisation dependent on the diagnoses and type of fall [33]. For instance, an examination of child injury hospitalisations across the SES gradient indicated that differences in rates only reached significance for falls from a low height [33].

In terms of child injury prevention, adverse home and neighbourhood structural conditions can elevate the risk of other forms of childhood injury. For example, children residing in more socioeconomically disadvantaged areas often live in older homes, have limited safe spaces and increased exposure to traffic, all of which elevate their risk of poisoning, burns and pedestrian injuries [36, 37]. Moreover, adverse home and neighbourhood structures are also marked with low social cohesion, increased crime rates and poverty, which may explain the consistent pattern between high SES disadvantage and increased assault-related injury risk [38].

The causal mechanisms underlying the SES differences in childhood injury are complex and multi-factorial. The current findings support the influence of SES inequality on child injury morbidity for some types of injuries [39]. Designing interventions to target risk and protective factors of injuries is an important challenge for policy makers to reduce the burden of childhood injury among socioeconomically disadvantaged groups. Isolated or region specific interventions, such as parental education and home visitations for at risk mothers, have demonstrated positive short-term outcomes of reduced injuries among infants [40]. Improving medical literacy and provision of safety equipment has been shown to improve poison prevention practices, however, the impact of such interventions, while promising, is currently unclear [41]. Scald and burn prevention has had positive results through the installation of hot water regulating devices and smoke alarms [37].

While encouraging behavioural modifications through community-based prevention programmes is highly favoured, these are not always effective for certain injuries, such as burns and pedestrian collisions [37, 42]. Compared to interventions focusing on individual behaviour change, interventions at a community-level focusing on both environmental and individual factors, such as safer housing designs in public or low-income housing, redesigning roads through installing speed bumps and reducing road hazards to allow safer crossing may be a more effective injury prevention strategy [5, 39, 43]. However, early intervention programs that target high risk and/or violent behaviour among youth remain an important challenge for policy makers to reduce the burden of intentional injuries.

The current study has several limitations. Due to changes in statistical area portioning of quintiles, trends of hospitalisation injury by SES quintile were unable to be estimated [27], and it is most likely that the sudden increase in injury hospitalisation rates between 2007 and 08 and 2009–10 for SES quintiles 1 and 2 reflects this change. There was an under-enumeration of total injury hospitalisations as there was no information on injury hospitalisations in the Australian Capital Territory prior to 1 July 2004 and up to 3975 injury hospitalisations each year in Victoria were unable to be linked. The present study relies on an area-based indicator of SES disadvantage, and may be subject to ecological fallacy [44]. As the IRSD summarises the characteristics of people and households within a geographical area, it does not reflect individual differences or specific households (e.g. low income does not always equate to disadvantage, as certain low-income households may have access to different social and economic resources that could help them mitigate their risk of childhood injury). Nonetheless, area-based classifications have been shown to correlate strongly with individual-level SES [45], and other studies have also found that area-based measures of SES disadvantage are associated with risk of childhood injury, independent of individual-level SES [45, 46]. It is possible that there is variation in the use of hospital services for children from different SES backgrounds; for example individuals may choose to use other available medical services to treat minor injury. Further research is needed to examine the type of health services used by injured children from different SES backgrounds.

## Conclusions

This is the first population-based epidemiological study examining hospitalised injury and SES disadvantage in Australian children over a 10-year period. The findings support the notion that the risk of hospitalisations from certain injury mechanisms, such as assault and pedestrian incidents, differs for children according to SES gradient. A national injury prevention strategy implementing interventions directed at modifying the built environment in conjunction with community and home interventions, will function as a way toward reducing childhood injury morbidity.

## Abbreviations

ICD-10-AM: International Classification of Diseases, 10th Revision, Australian Modification
ICISS: International Classification of Disease Injury Severity Score
IRR: Incidence rate ratio
IRSD: Index of relative socioeconomic disadvantage
SES: Socioeconomic status
SRR: Survival risk ratio
